# Intra-abdominal haemorrhage from uterine fibroids: a systematic review of the literature

**DOI:** 10.1186/s12893-020-00736-5

**Published:** 2020-04-15

**Authors:** Wei How LIM, Sally Charlotte COHEN, Vincent P LAMARO

**Affiliations:** Department of Gynaecology, St Vincent’s Hospital Sydney, 390 Victoria Street, Darlinghurst, New South Wales 2010 Australia

**Keywords:** Uterine leiomyomas, Intra-abdominal haemorrhage, Emergency laparotomy, haemoperitoneum

## Abstract

**Background:**

Uterine leiomyomas are common benign tumours found in women of reproductive age that are rarely associated with intra-abdominal haemorrhage. The aetiology behind this relationship is poorly understood and the aforementioned association poorly recognized from a patient’s clinical presentation. Available information in the literature is limited to case reports. The aim of this systematic review is to document and highlight the occurrence of intra-abdominal haemorrhage from uterine fibroids, and determine associated morbidity and mortality.

**Methods:**

A systematic review of Medline, EMBASE, Web of Science, Scopus, and The Cochrane Library – CENTRAL was performed from the databases inception through to December 2018 for case report and series of patients who experienced intra-abdominal haemorrhage from uterine fibroids. Findings were presented according to the Preferred Reporting Items for Systematic Reviews and Meta-Analysis (PRISMA) guidelines.

**Results:**

We identified 115 publications reporting on 125 original case reports. The documented intra-abdominal haemorrhage were commonly due to the rupture of superficial blood vessels over the surface of a fibroid, followed by rupture and avulsion of the fibroid involved. A clinical picture of sudden and profound hypovolemic shock with severe abdominal pain was often the presenting complaint, with a correct pre-operative diagnosis only made in 7 cases on computed tomography imaging. Hysterectomy and myomectomy were the most common surgery performed. Mortality was reported in 4 cases which were directly related to complications of uterine fibroids.

**Conclusion:**

Intra-abdominal haemorrhage secondary to uterine fibroids remained a rare phenomenon which is poorly recognized among clinicians. While this association is not representative of the population of interest, it highlights the pathophysiological spectrum of uterine fibroids and its relevance to emergency physicians, surgeons and gynaecologists during clinical practice.

## Background

Uterine fibroids or leiomyoma are benign smooth-muscle tumours resulting from the proliferation of myometrial cells [1, 2]. They are hypersensitive to estrogen, relatively avascular, with a blood supply arising from the pseudo-capsules of myometrial tissue, and are often surrounded by an abnormal venous plexus [1, 3]. These tumours are more prevalent in women of African descent and estimated to be clinically apparent in 25% of women of reproductive age [2]. Women aged 30–40 years are most symptomatic, with menstrual and reproductive morbidities warranting medical treatment [2, 4]. The location and size of fibroids in the uterus are important determinants of symptomatology and clinical manifestations.

Acute complications arising from leiomyoma are uncommon, and may include torsion of a subserosal or pedunculated fibroid, degenerative changes and intra-abdominal haemorrhage [2, 5]. The latter is a rare manifestation, given that most haemorrhage from uterine fibroids is vaginal, and is associated with high morbidity and mortality. The aetiology of this manifestation is poorly understood and hence poorly recognized from clinical presentation. Because of its rarity, a clinical picture of sudden and profound hypovolemic shock with severe abdominal pain often suggests other more common causes, including gastrointestinal and vascular disorders, necessitating emergency surgical intervention [2, 6]. The correct diagnosis is rarely identified before surgery, despite advanced medical imaging modalities.

The available information about intra-abdominal haemorrhage associated with uterine fibroids is limited to case reports and a handful of review articles of varying reporting styles, published between the 1950s and the 1970s [6–10]. To better understand this phenomenon, we conducted a systematic review of all identified cases of intra-abdominal haemorrhage from uterine fibroids in the literature to summarize current and updated evidence surrounding its management.

## Methods

A systematic review of cases identifying intra-abdominal haemorrhage from uterine leiomyoma was performed. We searched electronic databases (Medline, EMBASE, Web of Science, Scopus, The Cochrane Library – CENTRAL) from the databases inception through to December 2018 with the following key words: “intra-abdominal haemorrhage” OR “intra-peritoneal haemorrhage” OR “haemoperitoneum” AND “uterine fibroid” OR “leiomyoma” OR “myoma” OR “leiomyosarcoma”. Extra-uterine leiomyoma causes and other causes of abdominal haemorrhage were excluded. Only publications in the English language were included. Additional references and bibliographies of relevant articles included were located manually. All articles were obtained through Walter McGrath Library through our tertiary institution. No authors were contacted.

### Study selection

We included original individual case reports or series describing clinical presentation and subsequent management. The following types of articles were excluded: conference abstracts; case reports summarized in review articles; and incomplete case reports. Assessment of case report quality and findings were independently reviewed by two authors then entered into a Microsoft Excel spreadsheet database. Regular discussions between authors, either in person or through email, were carried out to resolve any disagreements. The following data were extracted from each case report: details of the author; year and country of publication; patient demographics; clinical presentation and findings; primary mode of intervention (medical or surgical); pathology results; surgical findings and outcome.

Statistical analysis was conducted using InStat version 3.1 (GraphPad Software, Inc., California, USA). As a result of study heterogeneity, a pooled descriptive analysis and narrative synthesis of the results was conducted. Data were summarized with means and standard deviations for continuous variables; frequencies and percentages were summarized for dichotomous variables. Categorical variables were compared using chi-square testing or Fisher’s exact test. All reported *p*-values are two-sided and *p* < 0.05 was considered significant.

**Fig. 1:**
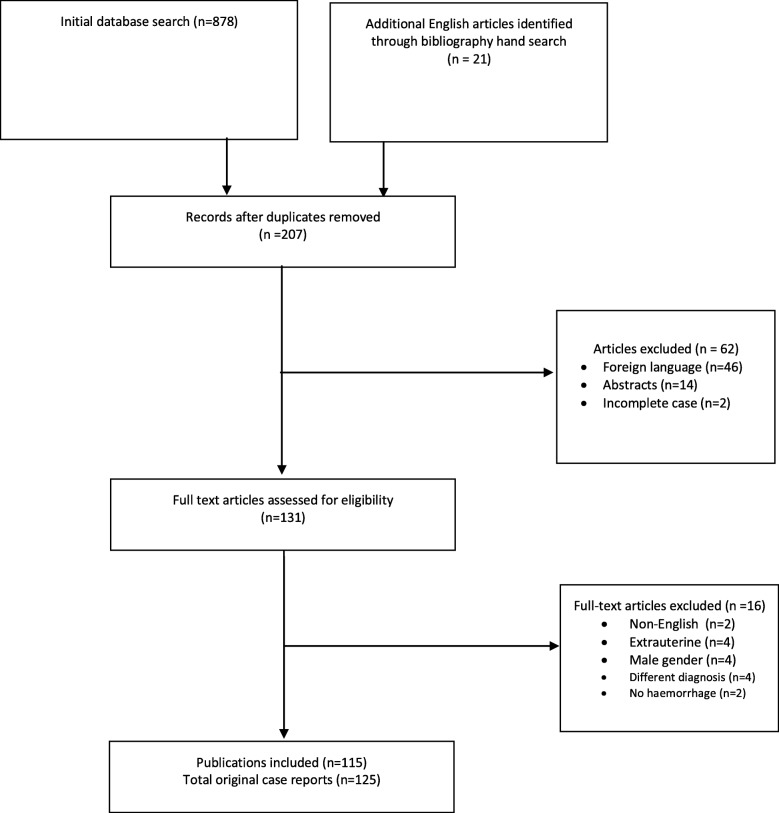
Preferred Reporting Items for Systematic Reviews and Meta-Analysis (PRISMA) flow chart of study

This systematic review was reported using the Preferred Reporting Items for Systematic Reviews and Meta-Analysis (PRISMA) guidelines [11]. Figure 1 illustrates the PRISMA flow diagram for study selection.

## Results

### Publication characteristics

Database searches retrieved a total of 878 citations. The search identified no randomized controlled trials, and no prospective or retrospective case-controlled studies. After removing duplicated studies and screening for language and individual case reports, 115 publications were identified that provided a clinical description for 125 original case reports. Full bibliographic references and quality appraisal assessment are available in Appendix 1. In 5 reports, haemorrhage occurred within the leiomyoma and these were also included in the review.

We divided the manuscripts into three 35-year periods to address a temporal trend: 1902 to 1940 had 19 case reports; 1941 to 1979, 22 cases; 1980 to 2018, 84 cases. The majority of these articles were from the United States (32.2%), followed by the United Kingdom (21.7%), Japan (6.4%) and India (6.4%). The clinical characteristics of all 125 reported cases are summarized in Table 1.

**Table 1:** Clinical characteristics of all 125 reported cases

Characteristics	***n*** = 125
Age (mean, SD)	40.5 (9.2)
Pre-menopausal, n (%)	104 (83.2)
Post-menopausal, n (%)	21 (16.8)
Parity, n (%)	
• Nulliparity	37 (29.6)
• Multiparity	48 (38.4)
Presentation, n (%)	
• Spontaneous	106 (84.8)
• Trauma	19 (15.2)
Symptoms, n (%)	
• Abdominal pain	124 (99.2)
• Syncope/collapse	27 (21.6)
• Vomiting & diarrhea	14 (11.2)
• Shoulder tip pain	11 (8.8)
• Dyspnea	8 (6.4)
• Fever	6 (4.8)
Antecedent to presentation, n (%)	
• Menstruation	15 (12)
• MVA/fall	11 (8.8)
• Stretching/exercise	8 (6.4)
• Defecation	6 (4.8)
Hypovolemic shock, n (%)	87 (69.6)
Cardiac arrest, n (%)	2 (1.6)
Mean size fibroid, cm (SD)	12.6 (6.4)
Types of fibroid, n (%)	
• Subserosal	47 (37.6)
• Pedunculated	24 (19.2)
• Intramural	5 (4)
Location of fibroid, n (%)	
• Fundal	58 (46.4)
• Posterior	24 (19.2)
• Anterior	11 (8.8)
Etiology of hemorrhage, n (%)	
• Ruptured vessel	76 (60.8)
• Ruptured fibroid	34 (27.2)
• Avulsion fibroid	10 (8)
• Intra-fibroid	5 (4)
Haemoperitoneum, mean mls (SD)	1959.0 (974.3)
Laparotomy, n (%)	122 (97.6)
• During Cesarean section	3 (2.4)
Laparoscopy, n (%)	2 (1.6)
Type of surgery, n (%)	
• Hysterectomy	73 (58.4)
• Myomectomy	45 (36)
• Ligation of vessels	4 (3.2)
• With adnexal surgery	42 (33.6)
RBC transfusion, units (SD)	5.8 (5.44)
FFP transfusion, units (SD)	3.2 (1.2)
Death, n (%)	4 (3.2)

*SD* standard deviation; *RBC* red blood cells; *FFP* fresh frozen plasma

### Clinical manifestations

A mean age of 40.5 years (standard deviation (SD), 9.2 years) was identified. Only 23 cases reported on ethnicity of the patients, with 10 Caucasian women, 8 women of African descent, and 5 Japanese women. Other demographic details such as body mass index, smoking status, medical and surgical history were not routinely recorded. Parity was reported only in 85 patients. There were 6 patients in the 1st-2nd trimester of pregnancy and 12 patients in the perinatal period (4 in the late 3rd trimester and 8 in the puerperium period).

Abdominal pain was the most common symptom described on initial presentation: 104/125 (83.2%) presented with sudden onset lower abdominal pain, while 20/125 (16%) experienced a gradual onset over 3–5 days. There were 81/125 (64.8%) patients found to be in hypovolemic shock on initial presentation, with another 6/125 (4.8%) patients who developed it after arrival. Collapse or syncopal episode was seen in 27/125 (21.6%) patients; the next most common presenting symptom was dyspnoea, followed by nausea, seizures and diarrhoea. Eleven patients (8.8%) experienced shoulder tip pain. Two patients arrived in hospital with cardiac arrest (see Mortality and Morbidity).

Overall, documentation of both history of presenting complaint and past medical history was poor in all 125 patients. Fifteen patients were menstruating at time of presentation. Eleven patients (8.8%) presented following motor vehicle accidents or a fall. Six patients (4.8%) presented immediately after defecation. Only 40/125 (32.7%) women had a history of uterine fibroids who were previously asymptomatic. Of these, 5 were being monitored with a plan for elective surgery for fibroids removal. A pelvic mass or an enlarged uterus was only reported in 28/125 (22%) of women during physical examination. Other case studies commented on the difficulty in examining the subjects, due either to a presentation of acute abdomen or active resuscitation.

Of the 4 pregnant patients in late 3rd trimester, 1 patient presented with symptoms and signs suggestive of pre-eclampsia and placental abruption, while another patient presented in labour with non-reassuring fetal status. Eight patients (6.4%) developed symptoms during the puerperium period, ranging from 15 min to 5 weeks post-delivery. Of these patients, 6 had vaginal deliveries and the rest caesarean sections.

### Investigations & Diagnosis

In 120/125 (96%) of reported cases, a pre-operative diagnosis of intra-abdominal haemorrhage of unknown origin was established with the incidental finding of a uterine fibroid. Differential diagnoses included ectopic pregnancy, ruptured ovarian cyst, perforated peptic ulcer and ruptured spleen. From 1902 to 1940 and 1941 to 1979, the diagnosis of intra-abdominal haemorrhage was made clinically, i.e. without the aid of medical imaging. An abdominal mass was frequently felt on examination. There were only 7 documented cases where culdocentesis was performed to confirm haemoperitoneum.

From 1980 to 2018, medical imaging was performed in 66/84 (78.5%) of all cases. Pelvic ultrasonography (US), done either portably by the bedside or through formal scanning, was performed in 29 patients (34.5%) on initial presentation, while 19 patients (22.6%) underwent computed tomography (CT) imaging instead. Sixteen patients had both US and CT imaging performed, as the cause behind their clinical presentation was unclear. Magnetic resonance imaging (MRI) was performed in 2 cases.

All imaging modalities reported findings of ascites or intra-abdominal haemorrhage with a pelvic mass. In the course of identifying the origin of the haemorrhage a number of CT scans reported on extravasation of contrast material. Of the 11 cases that specifically reported on contrast enhancement, 7 patients were diagnosed with active contrast extravasation from uterine fibroids.

### Aetiology and pathology

The most common cause of haemoperitoneum was the rupture of superficial blood vessels over the surface of a fibroid, identified in 76/125 (60.8%) of women, all of whom were diagnosed intra-operatively. Among these cases, 58/76 (76.3%) described the bleed as venous in origin, while 9/76 (11.3%) were arterial. The remaining 9/76(11.3%) did not distinguish between the two blood vessel type. Other causes of haemoperitoneum included ruptured fibroid in 34/125 (27.2%) and fibroid avulsion in 10/125(8%).

The majority of cases reported involved a single dominant fibroid, with a mean maximum diameter of 12.6 cm (SD 6.4). Articles comparing size of fibroid to uterine size according to weeks of gestation found the average size to be 16–30 weeks gestation. Most fibroids described were subserosal 47/125 (37.6%) or pedunculated 24/125 (19.2%) in anatomical location, while 58/125 (46.4%) were fundal in origin and 24/125 (19.2%) arose from the posterior uterine wall. Only 14 articles reported on the weight of surgical specimens. The average weight reported for the fibroid was 1496.9 g (SD 131.4); the average weight for the uterus was 1255.9 g (SD 2032.2).

In the majority of cases, histopathology documentation showed benign leiomyoma or fibromyoma, with red or hyaline degeneration changes seen in 21 cases. Other reported pathologies included 5 leiomyosarcomas, 3 symplastic (atypical) leiomyoma, 2 smooth muscle tumours of uncertain malignant potential (STUMP), 1 chorio-endothelioma and 1 uterine tumour resembling an ovarian sex-cord tumour.

Five cases of intra-leiomyoma or intra-tumour haemorrhage were identified. Three patients were post-menopausal. One case had concurrent rupture of fibroid, and another had rupture of vein through an intramural fibroid. Minimal haemoperitoneum was found at time of surgery for all 5 cases; an average of 1707.5 mls of blood was found within the fibroid. All 5 patients received total hysterectomies, with an average fibroid weight of 12 kg.

### Primary mode of intervention

All patients were managed surgically, with no cases managed medically alone or managed conservatively. Indications for surgery were massive intra-abdominal haemorrhage with hemodynamic instability. Time interval from hospital presentation to surgery was immediate, with some reports averaging 3–5 days following initial presentation.

Laparotomy was the most common surgical approach, with laparoscopy successfully performed in only 2 cases (1.6%). Laparoscopic surgery was attempted in 8 women, 6 of which were converted to open. Two cases received pre-operative uterine arterial embolization. Hysterectomy was performed in 73/125 (58.4%) cases, with myomectomy in 45/125 (36%) cases and ligation of haemorrhagic vessels in 4/125 (3.2%). Of those women who received a hysterectomy, 48/73 (65.7%) had total hysterectomies and the remaining were subtotal. Concurrent adnexal surgery (i.e. unilateral or bilateral salpingo-oophorectomy) was performed in 42/125 (33.6%) cases. Of the 4 pregnant patients presenting in their late 3rd trimester, 2 myomectomies and 1 total hysterectomy were performed in conjunction with their caesarean sections.

Myomectomy was the preferred intervention over hysterectomy when a ruptured vessel was identified over the uterine fibroid (74.4% vs 32.1%; *p* < 0.05). Patients who had a hysterectomy performed were generally older (44.1 vs 35.9; *p* < 0.05), were found to have a ruptured fibroid (40.8% vs 16.2%; p < 0.05) as source of bleeding, and had documented sarcomatous changes on pathology (11.2% vs 0%; p < 0.05) when compared to patients who had received a myomectomy. There were no differences in prevalence of shock, blood loss or transfusions between both groups.

### Morbidity & Mortality

The average volume of haemoperitoneum encountered during surgery was 1959 mls (SD 974.3). Older articles tend to report this finding as “large” or “full of blood”. The average unit of red blood cells transfused was 5.8 units (SD 5.44) and fresh frozen plasma transfusions, 3.2 units (SD 1.2). Complications from surgery were low with very few reported. There were 2 cases of post-operative infection with 1 case of abscess formation, 1 case of ileus, 1 case of pneumonia, and 1 case of thrombosis.

Mortality was reported in 4 cases, identified during the 1980 to 2018 time period. Three were directly related to hypovolemic shock – 2 patients presented with cardiac arrest and were not able to be resuscitated. One patient died of hypoxic brain injury on the 5th day post-surgery. One patient died from sepsis related fibroid degeneration. There were 2 other cases of mortality, separately attributed to complications secondary to chemotherapy and tumour metastases. Of the 10 patients who were pregnant, there were 2 miscarriages at 15 weeks gestation and 1 reported case of fetal demise at 38 weeks gestation with unknown cause the documented cause of death.

## Discussion

Intra-abdominal haemorrhage due to uterine fibroids is rare as evidenced by the identification of only 125 cases in the English-language literature since 1902. The 84 cases from 1980 to 2018 echo the challenges of modern practice, in that the clinical presentation is abrupt and difficult to manage. Women who develop this complication typically present in hypovolemic shock with abdominal pain without a clear pre-operative diagnosis prior to surgery, and in our review was found to have a mortality rate of 3.2%.

The source of haemorrhage in over half of all the patients identified was a ruptured superficial vessel overlying a leiomyoma. There is a historical scientific basis supporting abnormal vasculature to subserosal fibroids that is susceptible to spontaneous rupture. In 1913, John Sampson found that through injection studies on 150 fibroid uteri the subserous location of a fibroid significantly changed the vascular architecture of the uterus [12]. He demonstrated that a significant portion of the uterine arterial supply is shunted to the tumor, with venous drainage through dilated veins crossing over the surface of the fibroid and entering large channels at the periphery of the supporting myometrium. These vascular malformations give rise to prominent and weak superficial blood vessels and are associated with fibroids of 10 cm or more in diameter. A few authors have hypothesized that the posterior location of these fibroids predisposes patients to direct-contact injury from the promontory of the sacrum [8, 10].

A number of factors precipitating rupture of these superficial blood vessels or avulsion of a pedunculated fibroid were previously suggested [9]. These are based on the theory that increased blood flow or mechanical trauma would indirectly cause over-distention and tearing, leading to the onset of haemorrhage [6, 9]. These include venous congestion during menstruation, uterine manipulation (e.g. vaginal examination), increased venous pressure associated with defecation or lifting of heavy objects, and pregnancy. Trauma, such as falls, or motor vehicle accidents have also been linked to clinical presentation. These factors, however, were only identified in 26% of cases in our review, with 14% related to pregnancy or occurring post-partum. It is possible that due to the life-threatening nature of the presentation many of these factors were not explored either pre or post-surgery.

Causes of spontaneous rupture of uterine fibroid include degenerative and sarcomatous changes, leading to necrosis and perforation. Degenerative changes in fibroids are usually due to inadequate blood supply [2], and are nearly present in all tumours with no significant relationship between the presenting symptoms [13, 14]. These changes were seen in 35% of those who had ruptured fibroids in our review. Sepsis is one potential complication, and in our review led to one case of mortality. Environmental factors and hormonal therapy have been implicated in the pathogenesis of such changes [4], however these factors were again not routinely reported or explored in the case reports. All sarcoma and atypical leiomyoma pathology were later documented in women who had spontaneous rupture of uterine fibroid.

Fibroid avulsion – the separation of fibroid from the body of uterus – may occur spontaneously but is more common following an injury [9, 10]. This was noted in our review of 5 patients who presented following motor vehicle accidents; 2 after a fall and 1 after swimming activity. All fibroids were pedunculated with a narrow stalk attached to the surface of uterus.

We decided to include the 5 cases of intra-leiomyoma haemorrhage in this review as they share the same clinical characteristics and presentation of an intra-abdominal haemorrhage from a uterine fibroid. Two patients were identified in the immediate post-partum state. The authors hypothesized that involution of the uterus after delivery promoted compression of venous drainage, but not arterial flow [15, 16]. This would lead to a large amount of blood sequestration from maternal circulation into the fibroid, resulting in hypovolemia without haemoperitoneum.

A pre-operative diagnosis of intra-abdominal haemorrhage of unknown origin associated with pelvic mass was often made at clinical presentation. Under such circumstance, the presence of a uterine fibroid may be regarded as a “red herring”. A precise preoperative diagnosis is rarely made and our result of 5.3% is consistent with that of a previous review [9]. Pelvic US and MRI have no role in definite diagnosis; CT imaging with evidence of active contrast remains the best diagnostic modality available. It is important to emphasise that surgery should not be delayed, especially in a setting of profound hemodynamic instability.

Catastrophic intra-abdominal haemorrhage often prompts exploratory surgery even in the absence of a specialty-specific surgical team, and precludes consideration for alternate forms of management (e.g. uterine arterial embolization). The definitive management, however, is strictly surgical, including myomectomy or hysterectomy, with the primary aim of controlling life-endangering haemorrhage. Nevertheless, the surgeon needs to be vigilant about other causes of bleeding during diagnostic surgery. Teruo Risai reports a case of a 38-year-old woman who presented with sudden collapse and cardiac arrest [17]. An autopsy showed a 3-l haemoperitoneum with a large uterine leiomyoma (weight 2.5 kg); the documented cause of death was a ruptured splenic aneurysm. This emphasised the importance of a multi-disciplinary team of surgeons and gynaecologists, as well as access to blood bank and intensive care monitoring.

Our systematic review is based on a comprehensive literature search with a defined inclusion criteria and quality bibliographical search. However, each case report is limited by the quality of information available. Non-English language articles were excluded, leaving out potentially important information and further cases that could improve analysis. Mortality may have been under-reported, leading to publication bias. While uterine fibroids are common tumours in women of reproductive age, these rare associations are not representative of the population of interest. They do, however, highlight the pathophysiological spectrum of uterine fibroids and provide an important reference for this dangerous manifestation in clinical practice.

## Conclusion

Intra-abdominal haemorrhage of spontaneous or traumatic nature in women of reproductive age is commonly associated with ruptured ectopic pregnancy or ruptured corpus luteum [1, 2]. It is rarely a complication of uterine fibroids but deserves to be highlighted as a possible source of bleeding, especially in the presence of a pelvic mass detected during clinical examination and a negative pregnancy test. We propose a streamlined access to CT imaging with prompt resuscitative measurements, as well as a multidisciplinary operative approach among surgeons and gynaecologists to aid management and improve outcome. Prospective studies are necessary to address incidence and other evidence-based recommendations.

## Supplementary information

**Additional file 1: Appendix 1.** Full bibliographic references and quality appraisal assessment of all 125 reported cases.

## Data Availability

The datasets generated and analysed during the current study are available in supplementary document Appendix 1.

## Abbreviations

PRISMA: Preferred Reporting Items for Systematic Reviews and Meta-Analysis
US: Ultrasonography
CT: Computed computed tomography
MRI: Magnetic resonance imaging
STUMP: Smooth muscle tumours of uncertain malignant potential
