# Supplementary material for: Intra-abdominal haemorrhage from uterine fibroids: a systematic review of the literature
Source: BMC Surg. 2020 Apr 15;20:70. doi: 10.1186/s12893-020-00736-5 (PMC7157977; doi:10.1186/s12893-020-00736-5)
Supplement: Supplementary file 1 — Additional file 1: Appendix 1. Full bibliographic references and quality appraisal assessment of all 125 reported cases. [file 12893_2020_736_MOESM1_ESM.docx]

**Appendix 1. Full bibliographic references and quality appraisal assessment.**

| **Author** | **Year** | **Country** | **Original case report** | **Surgery described** | **Pathology identified** |
| --- | --- | --- | --- | --- | --- |
| Abdelazim^1^ | 2018 | Egypt | Yes | Yes | Yes |
| Adeyemi^2^ | 2012 | Nigeria | Yes | Yes | Yes |
| Ahltorp^3^ | 1934 | Sweden | Yes | Yes | Yes |
| Akahira^4^ | 1998 | Japan | Yes | Yes | Yes |
|  |  |  | Yes | Yes | Yes |
| Alharbi^5^ | 2013 | Saudi Arabia | Yes | Yes | Yes |
| Avola^6^ | 1963 | USA | Yes | Yes | Yes |
| Aydin^7^ | 2015 | Turkey | Yes | Yes | Yes |
| Badawy^8^ | 1961 | Egypt | Yes | Yes | Yes |
| Bangal^9^ | 2014 | India | Yes | Yes | Yes |
| Barua^10^ | 1998 | Australia | Yes | Yes | Yes |
| Bastu^11^ | 2012 | Turkey | Yes | Yes | Yes |
| Bell^12^ | 1990 | USA | Yes | Yes | Yes |
| Bigby^13^ | 1960 | UK | Yes | Yes | Yes |
| Blumenfeld^14^ | 1987 | Isreali | Yes | Yes | Yes |
|  |  |  | Yes | Yes | Yes |
| Bosch^15^ | 1950 | USA | Yes | Yes | Yes |
| Buttery^16^ | 1972 | Australia | Yes | Yes | Yes |
| Cerruto^17^ | 2016 | Italy | Yes | Yes | Yes |
| Chen^18^ | 2013 | Taiwan | Yes | Yes | Yes |
| Chetan^19^ | 2011 | UK | Yes | Yes | Yes |
| Chouduri^20^ | 1961 | India | Yes | Yes | Yes |
|  |  |  | Yes | Yes | Yes |
| Clarke^21^ | 1907 | UK | Yes | Yes | Yes |
| Collins^22^ | 1956 | USA | Yes | Yes | Yes |
| Culhaci^23^ | 2006 | Turkey | Yes | Yes | Yes |
| Dahan^24^ | 2002 | USA | Yes | Yes | Yes |
| Danikas^25^ | 1999 | USA | Yes | Yes | Yes |
| Dasari^26^ | 2005 | India | Yes | Yes | Yes |
| Davies^27^ | 1950 | UK | Yes | Yes | Yes |
|  |  |  | Yes | Yes | Yes |
| Davison^28^ | 2013 | USA | Yes | Yes | Yes |
| Deopuria^29^ | 1970 | UK | Yes | Yes | Yes |
| Divagno^30^ | 1996 | Italy | Yes | Yes | Yes |
| Drutman^31^ | 1992 | USA | Yes | Yes | Yes |
| Duffy^32^ | 1960 | USA | Yes | Yes | Yes |
| Ekane^33^ | 2013 | Africa | Yes | Yes | Yes |
| Elbiss^34^ | 2006 | UK | Yes | Yes | Yes |
| Ernst^35^ | 1922 | Denmark | Yes | Yes | Yes |
|  |  |  | Yes | Yes | Yes |
| Estrade^36^ | 2010 | France | Yes | Yes | Yes |
| Ezeome^37^ | 2003 | Nigeria | Yes | Yes | Yes |
| Farhi^38^ | 1993 | Isreali | Yes | Yes | Yes |
| Ferguson^39^ | 1907 | UK | Yes | Yes | Yes |
| Fontanrensky^40^ | 2013 | France | Yes | Yes | Yes |
|  |  |  | Yes | Yes | Yes |
| Galani^41^ | 2010 | Greece | Yes | Yes | Yes |
| Grisaru^42^ | 1995 | Isreali | Yes | Yes | Yes |
| Gulati^43^ | 2016 | UK | Yes | Yes | Yes |
| Gupta^44^ | 2008 | India | Yes | Yes | Yes |
| Gupta^45^ | 2009 | UK | Yes | Yes | Yes |
| Guy^46^ | 1994 | UK | Yes | Yes | Yes |
| Hamilton^47^ | 1932 | Australia | Yes | Yes | Yes |
| Hasskarl^48^ | 1949 | USA | Yes | Yes | Yes |
| Hicks^49^ | 2010 | UK | Yes | Yes | Yes |
| Hicks^50^ | 2014 | USA | Yes | Yes | Yes |
| Horowitz^51^ | 2005 | Isreali | Yes | Yes | Yes |
| Ihama^52^ | 2008 | Japan | Yes | Yes | Yes |
| Jain^53^ | 2004 | UK | Yes | Yes | Yes |
| Jenayah^54^ | 2017 | Tunisia | Yes | Yes | Yes |
| Kaavya^55^ | 2016 | India | Yes | Yes | Yes |
| Kamat^56^ | 2001 | India | Yes | Yes | Yes |
| Kassenge^57^ | 2017 | Togo | Yes | Yes | Yes |
| Kasum^58^ | 2010 | Croatia | Yes | Yes | Yes |
| Kaye^59^ | 1950 | USA | Yes | Yes | Yes |
| Kelson^60^ | 2018 | USA | Yes | Yes | Yes |
| Koide^61^ | 2009 | Japan | Yes | Yes | Yes |
| LaCoursiere^62^ | 2005 | USA | Yes | Yes | Yes |
| Lazaro^63^ | 1980 | USA | Yes | Yes | Yes |
| Lewers^64^ | 1905 | UK | Yes | Yes | Yes |
| Li^65^ | 1952 | USA | Yes | Yes | Yes |
| Littler^66^ | 1910 | UK | Yes | Yes | Yes |
| Lotterman^67^ | 2008 | USA | Yes | Yes | Yes |
| Luker^68^ | 1927 | UK | Yes | Yes | Yes |
| Makar^69^ | 1989 | Belgium | Yes | Yes | Yes |
| Manopunya^70^ | 2013 | Thailand | Yes | Yes | Yes |
| Manoukian^71^ | 2018 | USA | Yes | Yes | Yes |
| Mattison^72^ | 1980 | USA | Yes | Yes | Yes |
|  |  |  | Yes | Yes | Yes |
| McNeil^73^ | 1952 | UK | Yes | Yes | Yes |
| Mistry^74^ | 2016 | UK | Yes | Yes | Yes |
| Mizrahi^75^ | 2017 | USA | Yes | Yes | Yes |
| Nemer^76^ | 2014 | USA | Yes | Yes | Yes |
| Nolan^77^ | 1996 | USA | Yes | Yes | Yes |
| Oda^78^ | 2017 | Japan | Yes | Yes | Yes |
| Olagundoye^79^ | 2004 | UK | Yes | Yes | Yes |
| Peng^80^ | 2015 | Taiwan | Yes | Yes | Yes |
| Pereira^81^ | 2012 | USA | Yes | Yes | Yes |
| Prior^82^ | 2010 | UK | Yes | Yes | Yes |
| Ramskill^83^ | 2014 | UK | Yes | Yes | Yes |
| Ransohoff^84^ | 1921 | USA | Yes | Yes | Yes |
|  |  |  | Yes | Yes | Yes |
| Raslan^85^ | 1997 | UK | Yes | Yes | Yes |
| Robinson^86^ | 1996 | USA | Yes | Yes | Yes |
| Sadlier^87^ | 1913 | USA | Yes | Yes | Yes |
| Saidi^88^ | 1961 | USA | Yes | Yes | Yes |
| Schneider^89^ | 1942 | USA | Yes | Yes | Yes |
| Schwartz^90^ | 2017 | USA | Yes | Yes | Yes |
| Schwitkis^91^ | 2017 | USA | Yes | Yes | Yes |
| Seet^92^ | 2014 | Singapore | Yes | Yes | Yes |
| Seiji^93^ | 2005 | Japan | Yes | Yes | Yes |
| Shapira^94^ | 1932 | USA | Yes | Yes | Yes |
|  |  |  | Yes | Yes | Yes |
| Sheehan^95^ | 1951 | Ireland | Yes | Yes | Yes |
| Shelfo^96^ | 1939 | USA | Yes | Yes | Yes |
|  |  |  | Yes | Yes | Yes |
| Shelley^97^ | 1931 | USA | Yes | Yes | Yes |
| Siewert^98^ | 2007 | USA | Yes | Yes | Yes |
| Su^99^ | 2007 | Taiwan | Yes | Yes | Yes |
| Sule^100^ | 2000 | Nigeria | Yes | Yes | Yes |
| Swarray^101^ | 2017 | Ghanna | Yes | Yes | Yes |
| Tajima^102^ | 2015 | Japan | Yes | Yes | Yes |
| Takai^103^ | 2013 | Japan | Yes | Yes | Yes |
| Tan^104^ | 2014 | Malaysia | Yes | Yes | Yes |
| Tang^105^ | 2009 | UK | Yes | Yes | Yes |
| Tirelli^106^ | 2008 | Italy | Yes | Yes | Yes |
| Toquero^107^ | 2011 | UK | Yes | Yes | Yes |
| Turner^108^ | 1920 | UK | Yes | Yes | Yes |
| Varras^109^ | 2002 | Greece | Yes | Yes | Yes |
| Venebles^110^ | 1967 | UK | Yes | Yes | Yes |
| Von Son^111^ | 1965 | USA | Yes | Yes | Yes |
| Wallace^112^ | 1910 | UK | Yes | Yes | Yes |
| Woodruff^113^ | 1948 | UK | Yes | Yes | Yes |
| Wong^114^ | 2005 | Singapore | Yes | Yes | Yes |
| Wyckoff^115^ | 2016 | USA | Yes | Yes | Yes |

1. Abdelazim IA, Abu-Faza M, Zhurabekova G, et al. [Intra-leiomyoma hemorrhage in postmenopausal woman presented with acute abdominal pain.](https://www.ncbi.nlm.nih.gov/pubmed/30598976) J Family Med Prim Care. 2018 Sep-Oct;7(5):1129-1132
2. [Adeyemi](https://www.liebertpub.com/doi/10.1089/gyn.2010.0082) AB, [Bolaji](https://www.liebertpub.com/doi/10.1089/gyn.2010.0082) OF, [Aworinde](https://www.liebertpub.com/doi/10.1089/gyn.2010.0082) OO, et al. [Acute Cardiovascular Collapse Secondary to Massive Hemoperitoneum from a Bleeding Uterine Fibroid](https://www.liebertpub.com/doi/full/10.1089/gyn.2010.0082). J Gynecol Surg 2012;28:40-42
3. Ahltorp G. On spontaneous rupture of the myoma capsule. Acta Obstet Gynecol Scand. 1934;14:368-381
4. Akahira J, Ito K, Nakamura R, Yajima A. [Massive HYPERLINK "https://www.ncbi.nlm.nih.gov/pubmed/9823782"intraperitonealHYPERLINK "https://www.ncbi.nlm.nih.gov/pubmed/9823782" HYPERLINK "https://www.ncbi.nlm.nih.gov/pubmed/9823782"hemorrhageHYPERLINK "https://www.ncbi.nlm.nih.gov/pubmed/9823782" and HYPERLINK "https://www.ncbi.nlm.nih.gov/pubmed/9823782"hypovolemicHYPERLINK "https://www.ncbi.nlm.nih.gov/pubmed/9823782" shock dueHYPERLINK "https://www.ncbi.nlm.nih.gov/pubmed/9823782" to rupture of a coronary vessel of a uterine HYPERLINK "https://www.ncbi.nlm.nih.gov/pubmed/9823782"leiomyomaHYPERLINK "https://www.ncbi.nlm.nih.gov/pubmed/9823782": HYPERLINK "https://www.ncbi.nlm.nih.gov/pubmed/9823782"aHYPERLINK "https://www.ncbi.nlm.nih.gov/pubmed/9823782" report of two cases.](https://www.ncbi.nlm.nih.gov/pubmed/9823782) Tohoku J Exp Med. 1998 Jul;185(3):217-22.
5. Alharbi SR. Uterine leiomyoma with spontaneous intraleiomyoma hemorrhage, perforation, and hemoperitoneum in postmenopausal woman: Computed tomography diagnosis. Avicenna J Med 2013;3:81-83.
6. AvolA FA, Gonnella JP. [Intraperitoneal Hemorrhage due to uterine fibroid.HYPERLINK "https://www.ncbi.nlm.nih.gov/pubmed/14056250" HYPERLINK "https://www.ncbi.nlm.nih.gov/pubmed/14056250"Report of a case in late pregnancy.](Intraperitoneal%20Hemorrhage%20due%20to%20uterine%20fibroid.%20Report%20of%20a%20case%20in%20late%20pregnancy.) Arch Surg. 1963 Oct;87:666-8.
7. Aydin C, Sen Selim H, Eriş S, et al. Haemoperitoneum: an extremely rare complication of leiomyoma. J Obstet Gynaecol 2015;35:109-110.
8. Badawy AH. [Diffuse HYPERLINK "https://www.ncbi.nlm.nih.gov/pubmed/13685733"intraperitonealHYPERLINK "https://www.ncbi.nlm.nih.gov/pubmed/13685733" haemorrhage from surface vein of a HYPERLINK "https://www.ncbi.nlm.nih.gov/pubmed/13685733"fibromyomaHYPERLINK "https://www.ncbi.nlm.nih.gov/pubmed/13685733".](https://www.ncbi.nlm.nih.gov/pubmed/13685733) Br Med J. 1961 Mar 11;1(5227):717-8.
9. Bangal VB, Shinde KK, Gavhane SP, et al. Massice hemoperitoneum due to rupture of surface veins of a uterine leiomyoma. International Journal of Biomedical And Advance Research. 2014;05(02):128-130
10. Barua R, Olesnicky G. [Uterine leiomyosarcoma presenting with haemoperitoneum.](file:///I:\pubmed\3380046) Med J Aust. 1988 Jun 20;148(12):655, 657.
11. Bastu E, Akhan SE, Ozsurmeli M, Galandarov R, Sozen H, Gungor-Ugurlucan F, Iyibozkurt AC. [Acute hemorrhage related to spontaneous rupture of an uterine fibroid: a rare case report.](https://www.ncbi.nlm.nih.gov/pubmed/23967563) Eur J Gynaecol Oncol. 2013;34(3):271-2.
12. Bell MH. [Intra-abdominal hemorrhage and shock due to uterine fibroid.](https://www.ncbi.nlm.nih.gov/pubmed/2314373) Mo Med. 1990 Mar;87(3):155-7.
13. Bigby MA. [IntraperitonealHYPERLINK "https://www.ncbi.nlm.nih.gov/pubmed/13800627" haemorrhage from a vessel on the surface of a uterine fibroid.](https://www.ncbi.nlm.nih.gov/pubmed/13800627) Br Med J. 1960 Aug 20;2(5198):580-1.
14. Blumenfeld D, Timor I, Shalev E. Intra-abdominal haemorrhage due to the rupture of a blood vessel overlying a uterine fibromyoma. J Obstet Gynaecol. 1987;8(2):184-185
15. Bosch DT, Carter OB, O'Grady MJ. [Massive HYPERLINK "https://www.ncbi.nlm.nih.gov/pubmed/15409868"intraperitonealHYPERLINK "https://www.ncbi.nlm.nih.gov/pubmed/15409868" HYPERLINK "https://www.ncbi.nlm.nih.gov/pubmed/15409868"hemorrhageHYPERLINK "https://www.ncbi.nlm.nih.gov/pubmed/15409868" from a ruptured coronary vessel of a uterine HYPERLINK "https://www.ncbi.nlm.nih.gov/pubmed/15409868"leiomyomaHYPERLINK "https://www.ncbi.nlm.nih.gov/pubmed/15409868", with report of a case.](https://www.ncbi.nlm.nih.gov/pubmed/15409868) Am Pract Dig Treat. 1950 Feb;1(2):177.
16. Buttery BW. [Spontaneous HYPERLINK "https://www.ncbi.nlm.nih.gov/pubmed/4511750"haemoperitoneumHYPERLINK "https://www.ncbi.nlm.nih.gov/pubmed/4511750" complicating uterine HYPERLINK "https://www.ncbi.nlm.nih.gov/pubmed/4511750"fibromyomaHYPERLINK "https://www.ncbi.nlm.nih.gov/pubmed/4511750".](https://www.ncbi.nlm.nih.gov/pubmed/4511750) Aust N Z J Obstet Gynaecol. 1972 Aug;12(3):210-3.
17. Cerruto E, Sudano MC, Ettore C, La Greca G, La Greca MG. [Difficult diagnosis of hemoperitoneum in a patient with a pelvic mass of large size.](https://www.ncbi.nlm.nih.gov/pubmed/27497942) Int J Surg Case Rep. 2016;26:197-8.
18. Chen CH, Lin JY, Tzeng CR, Chiu LH, Liu WM. Hemoperitoneum secondary to rupture of a superficial uterine artery overlying a subserosal myoma with no predisposing factors in a young woman. Taiwan J Obstet Gynecol 2013;52:133- 134.
19. Chetan UR, Sengupta S, Abdo K, et al. Haemoperitoneum due to ruptured subserous uterine leiomyoma. Gynecol Surg 2011;8:365.
20. Choudhuri PK. [IntraperitonealHYPERLINK "https://www.ncbi.nlm.nih.gov/pubmed/13693301" haemorrhage from fibroids.](https://www.ncbi.nlm.nih.gov/pubmed/13693301) Br Med J. 1961 Sep 2;2(5252):650.
21. Clarke WB. Intraperitoneal bleeding from a uterine fibroid, with acute distension of the abdomen; Abdominal section; Removal of the fibroid; Recovery. The Lancet. 1907;169(4349):8-9
22. [Collins D](https://www.ncbi.nlm.nih.gov/pubmed/?term=COLLINS%20D%5BAuthor%5D&cauthor=true&cauthor_uid=13313618). Spontaneous fibroid enucleation causing postpartum intraperitoneal hemorrhage. [Am J Obstet Gynecol.](https://www.ncbi.nlm.nih.gov/pubmed/13313618) 1956 May;71(5):1130-1.
23. Culhaci N, Ozkara E, Yüksel H, et al. [Spontaneously ruptured uterine angioleiomyoma.](https://www.ncbi.nlm.nih.gov/pubmed/16554917) Pathol Oncol Res. 2006;12(1):50-1.
24. Dahan MH, Ahmadi R. [Spontaneous HYPERLINK "https://www.ncbi.nlm.nih.gov/pubmed/12063882"subseHYPERLINK "https://www.ncbi.nlm.nih.gov/pubmed/12063882"rosalHYPERLINK "https://www.ncbi.nlm.nih.gov/pubmed/12063882" venous rupture overlying a uterine HYPERLINK "https://www.ncbi.nlm.nih.gov/pubmed/12063882"leiomyomaHYPERLINK "https://www.ncbi.nlm.nih.gov/pubmed/12063882". HYPERLINK "https://www.ncbi.nlm.nih.gov/pubmed/12063882"AHYPERLINK "https://www.ncbi.nlm.nih.gov/pubmed/12063882" case report.](https://www.ncbi.nlm.nih.gov/pubmed/12063882) J Reprod Med. 2002 May;47(5):419-20.
25. Danikas D, Theodorou SJ, Kotrotsios J, Sills C, Cordero PE. [HemoperitoneumHYPERLINK "https://www.ncbi.nlm.nih.gov/pubmed/10597071" from spontaneous bleeding of a uterine HYPERLINK "https://www.ncbi.nlm.nih.gov/pubmed/10597071"leiomyomaHYPERLINK "https://www.ncbi.nlm.nih.gov/pubmed/10597071": HYPERLINK "https://www.ncbi.nlm.nih.gov/pubmed/10597071"aHYPERLINK "https://www.ncbi.nlm.nih.gov/pubmed/10597071" case report.](https://www.ncbi.nlm.nih.gov/pubmed/10597071) Am Surg. 1999 Dec;65(12):1180-2.
26. Dasari P, Maurya DK. Hemoperitoneum associated with fibroid uterus. J Obstet Gynecol India 2005;55(6):553-554
27. Davies V. [Two cases of spontaneous HYPERLINK "https://www.ncbi.nlm.nih.gov/pubmed/15422430"intraperitonealHYPERLINK "https://www.ncbi.nlm.nih.gov/pubmed/15422430" haemorrhage from the superficial veins of a uterine HYPERLINK "https://www.ncbi.nlm.nih.gov/pubmed/15422430"fibromyomaHYPERLINK "https://www.ncbi.nlm.nih.gov/pubmed/15422430".](https://www.ncbi.nlm.nih.gov/pubmed/15422430) J Obstet Gynaecol Br Emp. 1950 Apr;57(2):248-50.
28. Davison JZ, Bennett TA, Jaffe IM. [Laparoscopic HYPERLINK "https://www.ncbi.nlm.nih.gov/pubmed/24050034"myomectomyHYPERLINK "https://www.ncbi.nlm.nih.gov/pubmed/24050034" for HYPERLINK "https://www.ncbi.nlm.nih.gov/pubmed/24050034"hemoperitoneumHYPERLINK "https://www.ncbi.nlm.nih.gov/pubmed/24050034" from a uterine HYPERLINK "https://www.ncbi.nlm.nih.gov/pubmed/24050034"leiomyomaHYPERLINK "https://www.ncbi.nlm.nih.gov/pubmed/24050034" with concomitant tubal abortion: a case report.](Laparoscopic%20myomectomy%20for hemoperitoneum from%20a%20uterine leiomyoma with%20concomitant%20tubal%20abortion:%20a%20case%20report.) J Reprod Med. 2013 Sep-Oct;58(9-10):438-40.
29. Deopuria RH, Grijalvo GB. Haemoperitoneum from ruptured varicose vein on surface of a fibroid uterus. J Obstet Gynecol India 1970;20:414-415
30. [Di Vagno G](https://www.ncbi.nlm.nih.gov/pubmed/?term=Di%20Vagno%20G%5BAuthor%5D&cauthor=true&cauthor_uid=8798319), [Cormio G](https://www.ncbi.nlm.nih.gov/pubmed/?term=Cormio%20G%5BAuthor%5D&cauthor=true&cauthor_uid=8798319), [Resta L](https://www.ncbi.nlm.nih.gov/pubmed/?term=Resta%20L%5BAuthor%5D&cauthor=true&cauthor_uid=8798319), et al. Uterine tumour resembling an ovarian sex-cord tumour presenting with spontaneous haemoperitoneum in pregnancy. [Aust N Z J Obstet Gynaecol.](https://www.ncbi.nlm.nih.gov/pubmed/8798319) 1996 May;36(2):213-5.
31. Drutman J, Fruechte DM. [HemoperitoneumHYPERLINK "https://www.ncbi.nlm.nih.gov/pubmed/1590170" due to traumatic avulsion of a HYPERLINK "https://www.ncbi.nlm.nih.gov/pubmed/1590170"pedunculatedHYPERLINK "https://www.ncbi.nlm.nih.gov/pubmed/1590170" uterine HYPERLINK "https://www.ncbi.nlm.nih.gov/pubmed/1590170"leiomyomaHYPERLINK "https://www.ncbi.nlm.nih.gov/pubmed/1590170".](https://www.ncbi.nlm.nih.gov/pubmed/1590170) AJR Am J Roentgenol. 1992 Jun;158(6):1410.
32. Duffy LT. [Intraperitoneal hemorrhage from a ruptured coronary vein of a uterine myoma.](https://www.ncbi.nlm.nih.gov/pubmed/13818470) Obstet Gynecol. 1960 Jun;15:746-7
33. Ekane GH, Tebeu PM, Obinchemti TE, et al. [Postpartum hemoperitoneum due to rupture of a blood vessel on a uterine pseudo tumor: a case report.](https://www.ncbi.nlm.nih.gov/pubmed/24672628) Pan Afr Med J. 2013 Oct 17;16:57
34. Elbiss HM, Neale E. [Uterine leiomyosarcoma mimicking a ruptured aortic aneurysm.](file:///I:\pubmed\16390730) J Obstet Gynaecol. 2006 Jan;26(1):85-6. No abstract available.
35. Ernst P, Gammeltoft SA. Two cases of fibromyoma with intraabdominal hemorrhage. Acta Obstet Gynecol Scand. 1922;1(1):104-126
36. Estrade-Huchon S, Bouhanna P, Limot O, Fauconnier A, Bader G. Severe life-threatening hemoperitoneum from posttraumatic avulsionof a pedunculated uterine leiomyoma. J Minim Invasive Gynecol 2010;17:651-652.
37. Ezeome IV, Ikem AC. Acute Haemoperitoneum from Ruptured Veins on a Leiomyomatous Uterus. Trop J Obstet Gynaecol, 2003, 20: 164-166
38. Farhi J, Dicker D, Ben-Rafael Z. [Leiomyosarcoma presenting as spontaneous hemoperitoneum.](file:///I:\pubmed\8096478) Int J Gynaecol Obstet. 1993 Mar;40(3):249-50.
39. Ferguson GB. Chorio-endothelioma of uterus; Intraperitoneal hemorrhage; Hysterectomy; Death. Transactions of the Obstetrical Society of London. 1907;49:57-62
40. Fontarensky M, Cassagnes L, Bouchet P, et al. Acute complications of benign uterine leiomyomas:treatment of intraperitoneal haemorrhage by embolisation of the uterine arteries. Diagn Interv Imaging 2013;94:885-890.
41. Galani P, Kapetanakis S, Papadopoulos C, et al. [Hypovolemic shock due to giant uterus leiomyoma detachment.](https://www.ncbi.nlm.nih.gov/pubmed/21265397) Akush Ginekol (Sofiia). 2010;49(5):68-71.
42. Grisaru D, Bar-Am A, Lessing JB. [Spontaneous perforation of a degenerative nongestational uterine leiomyoma.](https://www.ncbi.nlm.nih.gov/pubmed/8677127) Obstet Gynecol. 1996 May;87(5 Pt 2):882
43. Gulati N, Raman S, Srinivasan M, et al. Rare gynaecological emergency: massive intraperitoneal haemorrhage from spontaneous rupture of a superficial vessel on a large leiomyoma. BMJ Case Rep 2016;2016:bcr2015212576.
44. Gupta N, Dadhwal V, Misra R, et al. Atypical presentation of a leiomyoma as spontaneous massive haemoperitoneum. Eur J Obstet Gynecol Reprod Biol 2008;138:120-21
45. Gupta S, Manyonda IT. Acute complications of fibroids. Best Pract Res Clin Obstet Gynaecol 2009;23:609-617.
46. Guy PS, Phillips WDP. Uterine fibroids: an unusual cause of haemoperitoneum. J Obstet Gynaecol. 1994;14(3):211-212,
47. Hamilton I. Fibromyoma of the uterus causing intraperitoneal haemorrhage. MJA. 1932 Feb;1(7):235-6
48. Hasskarl WF. [IntraperitonealHYPERLINK "https://www.ncbi.nlm.nih.gov/pubmed/18118073" HYPERLINK "https://www.ncbi.nlm.nih.gov/pubmed/18118073"hemorrhageHYPERLINK "https://www.ncbi.nlm.nih.gov/pubmed/18118073" from the coronary vessels of a uterine HYPERLINK "https://www.ncbi.nlm.nih.gov/pubmed/18118073"leiomyomaHYPERLINK "https://www.ncbi.nlm.nih.gov/pubmed/18118073".](https://www.ncbi.nlm.nih.gov/pubmed/18118073) Proc Staff Meet Mayo Clin. 1949 Apr 13;24(8):207-11.
49. Hicks G, McCallum IJD, Ogah K, et al. Spontaneous uterine perforation secondary to uterine leiomyosarcoma presenting as acute abdomen with haemoperitoneum. J Obstet Gynaecol. 2010;30(2):211-212.
50. Hicks CW, Garcia L, Howley I, et al. Traumatic hemoperitoneum. JAMA Surg 2014;149:615-616.
51. Horowitz E, Dekel A, Feldberg D, et al. Massive hemoperitoneum due to rupture of an artery overlying a uterine leiomyoma: a case report. Acta Obstet Gynecol Scand 2005;84:408-409.
52. Ihama Y, Miyazaki T, Fuke C. Hemoperitoneum due to rupture of a subserosal vein overlying a uterine leiomyoma. Am J Forensic Med Pathol 2008;29:177-180.
53. Jain P, Pradhan P, Cietak KA, et al. [Acute abdomen following spontaneous HYPERLINK "https://www.ncbi.nlm.nih.gov/pubmed/15369956"varicealHYPERLINK "https://www.ncbi.nlm.nih.gov/pubmed/15369956" rupture overlying uterine HYPERLINK "https://www.ncbi.nlm.nih.gov/pubmed/15369956"leiomyomaHYPERLINK "https://www.ncbi.nlm.nih.gov/pubmed/15369956".](https://www.ncbi.nlm.nih.gov/pubmed/15369956) J Obstet Gynaecol. 2004 Aug;24(5):589.
54. Jenayah AA, Saoudi S, Sferi N, et al. Spontaneous subserosal venous rupture overlying a uterine leiomyoma in a young woman. Pan Afr Med J 2017;28:205.
55. Kaavya M, Saraswathi K. Laceration of Veins of Subserous Uterine Fibroid Presenting as Haemoperitoneum - A Case Report. Research Journal of Pharmaceutical, Biological and Chemical Sciences. 2016;7(5):2214-18
56. Kamat NV, Telkar HB, Ramani SK, et al. [Ruptured degenerated uterine fibroid diagnosed by imaging.](https://www.ncbi.nlm.nih.gov/pubmed/11704222) Obstet Gynecol. 2001 Nov;98(5 Pt 2):961-3.
57. Kassegne I, Kolani K, Tchangai B, et al. Myomectomies for massive hemoperitoneum from spontaneous bleeding of a uterine myoma. J Surgical Case Reports, 2017;7,1–3
58. Kasum M. Hemoperitoneum caused by a bleeding myoma in pregnancy. Acta Clin Croat 2010;49:197-200.
59. Kaye BB, Ficarra BJ. [Rupture of uterine vessel by twisted fibroid causing intra-abdominal HYPERLINK "https://www.ncbi.nlm.nih.gov/pubmed/14774145"hemorrhageHYPERLINK "https://www.ncbi.nlm.nih.gov/pubmed/14774145".](https://www.ncbi.nlm.nih.gov/pubmed/14774145) J Am Med Assoc. 1950 Oct 21;144(8):616-7.
60. [Kelson KR](https://www.ncbi.nlm.nih.gov/pubmed/?term=Kelson%20KR%5BAuthor%5D&cauthor=true&cauthor_uid=30443616), [Riscinti M](https://www.ncbi.nlm.nih.gov/pubmed/?term=Riscinti%20M%5BAuthor%5D&cauthor=true&cauthor_uid=30443616), [Secko M](https://www.ncbi.nlm.nih.gov/pubmed/?term=Secko%20M%5BAuthor%5D&cauthor=true&cauthor_uid=30443616). Point-of-care Ultrasonography of a Rare Cause of Hemoperitoneum. [Clin Pract Cases Emerg Med.](https://www.ncbi.nlm.nih.gov/pubmed/?term=kelson+fibroid) 2018 Sep 5;2(4):320-322.
61. Koide K, Sekizawa A, Nakamura M, et al. [Hypovolemic shock due to massive edema of a pedunculated uterine myoma after delivery.](https://www.ncbi.nlm.nih.gov/pubmed/19751345) J Obstet Gynaecol Res. 2009 Aug;35(4):794-6
62. LaCoursiere DY, Chin HG. Pedunculated Atypical Leiomyoma Presenting as a Hemoperitoneum. J Gynecol Surg 2005;21:21
63. Lazaro NA, Batts JA Jr, Rishi A, et al. [Rupture of leiomyosarcoma uteri with hemoperitoneum clinically simulating ruptured ectopic pregnancy.](file:///I:\pubmed\7373602) J Reprod Med. 1980 Apr;24(4):174-6.
64. Lewers AHN. Fibro-cystic tumour of the uterus, laceration on its surface; free intraperitoneal haemorrhage. Transactions of the obstetrical society of London. 1905;47:261-263
65. [Li](javascript:void(0);) GG, Braden RG. Rupture of a subserous vein on the surface of a uterine myoma causing massive intraperitoneal hemorrhage. Am J Obstet Gynecol. 1952 May;63(5):1167-1168
66. Littler RM. Rupture of Uterine Myoma, Due to a Fall, with Intraperitoneal Hæmorrhage. J Obstet Gynaecol Br Emp. 1910 May;17(5):423-425
67. Lotterman S. Massive hemoperitoneum resulting from spontaneous rupture of uterine leiomyoma. Am J Emerg Med 2008;26:974.e1-e2.
68. [Luker SG](https://www.ncbi.nlm.nih.gov/pubmed/?term=Luker%20SG%5BAuthor%5D&cauthor=true&cauthor_uid=19986040). Fibromyoma of Uterus, Rupture of Capsule, with Protrusion of part of the Tumour, with Secondary Necrosis. [Proc R Soc Med.](https://www.ncbi.nlm.nih.gov/pubmed/19986040) 1927 Aug;20(10):1657-9.
69. Makar AP, Meulyzer PR, Vergote IB, et al. [A case report of unusual complication of myomatous uterus in pregnancy: spontaneous perforation of myoma after red degeneration.](https://www.ncbi.nlm.nih.gov/pubmed/2753197) Eur J Obstet Gynecol Reprod Biol. 1989 Jun;31(3):289-93.
70. Manopunya M, Tongprasert F, Sukpan K, Tongsong T. [Intra-HYPERLINK "https://www.ncbi.nlm.nih.gov/pubmed/22765641"leiomyomaHYPERLINK "https://www.ncbi.nlm.nih.gov/pubmed/22765641" massive HYPERLINK "https://www.ncbi.nlm.nih.gov/pubmed/22765641"hemorrhageHYPERLINK "https://www.ncbi.nlm.nih.gov/pubmed/22765641" after delivery.](https://www.ncbi.nlm.nih.gov/pubmed/22765641) J Obstet Gynaecol Res. 2013 Jan;39(1):355-8.
71. Manoukian MAC, Tembhekar AR, Medeiros SE. [Positive Seatbelt Sign with Avulsed Leiomyoma following Motor Vehicle Accident Leading to Hemoperitoneum.](https://www.ncbi.nlm.nih.gov/pubmed/30225150) Case Rep Emerg Med. 2018 Aug 26;2018:4251408.
72. Mattison DR, Yeh SY. [HemoperitoneumHYPERLINK "https://www.ncbi.nlm.nih.gov/pubmed/7352537" from rupture of a uterine vein overlying a HYPERLINK "https://www.ncbi.nlm.nih.gov/pubmed/7352537"leiomyomaHYPERLINK "https://www.ncbi.nlm.nih.gov/pubmed/7352537".](https://www.ncbi.nlm.nih.gov/pubmed/7352537) Am J Obstet Gynecol. 1980 Feb 1;136(3):415-6.
73. McNeil AT. [A further case of HYPERLINK "https://www.ncbi.nlm.nih.gov/pubmed/14928099"intraperitonealHYPERLINK "https://www.ncbi.nlm.nih.gov/pubmed/14928099" haemorrhage from rupture of a vein on the surface of a uterine HYPERLINK "https://www.ncbi.nlm.nih.gov/pubmed/14928099"fibromyomaHYPERLINK "https://www.ncbi.nlm.nih.gov/pubmed/14928099"; with a note on possible aetiology.](https://www.ncbi.nlm.nih.gov/pubmed/14928099) J Obstet Gynaecol Br Emp. 1952 Apr;59(2):252-3.
74. Mistry M, Bhatt M, Haque L, et al. [Degenerated uterine fibroid with rupture of the surface leading to haemoperitoneum.](https://www.ncbi.nlm.nih.gov/pubmed/27988218) Eur J Obstet Gynecol Reprod Biol. 2017 Apr;211:216-217.
75. Mizrahi DJ, Kaushik C, Adamo R. Hypovolemic shock and hemoperitoneum from spontaneous avulsion of a large pedunculated uterine leiomyoma. J Radiol Case Rep 2017;31:15-21.
76. Nemer LB, Gaudenti D, Schroeder ED, et al. Rupture of a Subserosal Vein Overlying a Leiomyoma Causing Hemoperitoneum. J Gynecol Surg 2014;30(6):367-369
77. Nolan DG, Byron JW. Massive Hemoperitoneum Associated With Leiomyoma Uteri. J Pelvic Surg [1996](https://journals.lww.com/jpelvicsurgery/toc/1996/02030);2(3):138-140
78. Oda H, Hirakawa H. [Spontaneous rupture of uterine smooth muscle tumour presenting acute abdominal pain and HYPERLINK "https://www.ncbi.nlm.nih.gov/pubmed/29298793"haemoperitoneumHYPERLINK "https://www.ncbi.nlm.nih.gov/pubmed/29298793".](https://www.ncbi.nlm.nih.gov/pubmed/29298793) BMJ Case Rep. 2018 Jan 3;2018. pii: bcr-2017-222806.
79. [Olagundoye V](https://www.ncbi.nlm.nih.gov/pubmed/?term=Olagundoye%20V%5BAuthor%5D&cauthor=true&cauthor_uid=15383119), [Jackson S](https://www.ncbi.nlm.nih.gov/pubmed/?term=Jackson%20S%5BAuthor%5D&cauthor=true&cauthor_uid=15383119), [Manek S](https://www.ncbi.nlm.nih.gov/pubmed/?term=Manek%20S%5BAuthor%5D&cauthor=true&cauthor_uid=15383119). Fatal septicaemia following rupture of a gangrenous fibroid. [BJOG.](https://www.ncbi.nlm.nih.gov/pubmed/?term=Olagundoye+fibroid) 2004 Oct;111(10):1141-2.
80. Peng CR, Chen CP, Wang KG, et al. Spontaneous rupture and massive hemoperitoneum from uterine leiomyomas and adenomyosis in a nongravid and unscarred uterus. Taiwan J Obstet Gynecol 2015;54:198-200.
81. Pereira N, Dormosh M, Mapow BC, et al. An Unusual Case of Acute Abdomen Related to a Large Pedunculated Leiomyoma. J Gynecol Surg 2012;28(5):355-358
82. Prior T, Byrne H, Lyons D. Fibroids as a cause of intraperitoneal haemorrhage. J Obstet Gynaecol 2010;30:209.
83. Ramskill N, Hameed A, Beebeejaun Y. [Spontaneous rupture of uterine leiomyoma during labour.](https://www.ncbi.nlm.nih.gov/pubmed/25199188) BMJ Case Rep. 2014 Sep 8;2014. pii: bcr2014204364.
84. Ransohoff JL, Dreyfoos M. Dangerous intraperitoneal haemorrhage from uterine fibroid. Surg Gynec & Obst. 1921;33:296-298
85. [Raslan F](https://www.ncbi.nlm.nih.gov/pubmed/?term=Raslan%20F%5BAuthor%5D&cauthor=true&cauthor_uid=15511938), [Gillmer MD](https://www.ncbi.nlm.nih.gov/pubmed/?term=Gillmer%20MD%5BAuthor%5D&cauthor=true&cauthor_uid=15511938). Spontaneous haemoperitoneum caused by subserous fibroid. [J Obstet Gynaecol.](https://www.ncbi.nlm.nih.gov/pubmed/?term=raslan+fibroid) 1997 Sep;17(5):496.
86. Robinson JN, Javeed M, Azoury R, et al. [Uterine Leiomyoma a cause of catastrophic intraperitoneal hemorrhage and death. J Pelvic Surg. 1996;2(5):271-273](https://journals.lww.com/jpelvicsurgery/Abstract/1996/09000/Uterine_Leiomyoma_A_Cause_of_Catastrophic.11.aspx)
87. Sadlier JE. Some complications of uterine fibroids demanding early diagnosis and immediate operation. Am J Obstet Dis Women Child. 1913;67(1):87-96
88. Saidi F, Constable JD, Ulfelder H. [Massive intraperitoneal hemorrhage due to uterine fibroids.](https://www.ncbi.nlm.nih.gov/pubmed/13745415) Am J Obstet Gynecol. 1961 Aug;82:367-74.
89. Schneider M, Jemerin EE. Intra-abdominal hemorrhage due to spontaneous rupture of a vein on a fibroid uterus. Am J Surg. 1942; 58: 294-295
90. Schwartz M, Powell K. Spontaneous rupture of a leiomyoma causing life-threatening intra-abdominal hemorrhage. Case Rep Obstet Gynecol 2017;2017:3701450.
91. Schwitkis A, Shen S, Vos E, et al. [Spontaneous Hemoperitoneum from Rupture of Massive Leiomyoma.](https://www.ncbi.nlm.nih.gov/pubmed/29849383) Clin Pract Cases Emerg Med. 2017 Mar 16;1(2):148-149
92. Seet MJ, Chonkar S, Mathur M. An unusual presentation of a ruptured degenerative fibroid in a perimenopausal woman. BMJ Case Rep 2014;2014:2014207473.
93. [Seiji M](https://www.ncbi.nlm.nih.gov/pubmed/?term=Seiji%20M%5BAuthor%5D&cauthor=true&cauthor_uid=16394937), [Shinnichi I](https://www.ncbi.nlm.nih.gov/pubmed/?term=Shinnichi%20I%5BAuthor%5D&cauthor=true&cauthor_uid=16394937), [Motojyuku M](https://www.ncbi.nlm.nih.gov/pubmed/?term=Motojyuku%20M%5BAuthor%5D&cauthor=true&cauthor_uid=16394937), et al. Traumatic avulsion of the uterine myoma. [J Trauma.](https://www.ncbi.nlm.nih.gov/pubmed/16394937) 2005 Dec;59(6):1532.
94. Shapira AA, Starr A. Massive Intraperitoneal Hemorrhage from Ruptured Subserous Veins on the Surface of Uterine Fibroids. N Engl J Med 1932; 207:827-829
95. Sheehan V. [Haemorrhage from coronary vessels of uterine myoma.](https://www.ncbi.nlm.nih.gov/pubmed/14897480) Ir J Med Sci. 1951 Jun;(306):285-8.
96. [Shelfo](javascript:void(0);) AL. Massive intraperitoneal hemorrhage resulting from rupture of a superficial vein of a uterine fibromyoma. [Am J Obstet Gynecol.](https://www.ncbi.nlm.nih.gov/pubmed/13313618) 1939 June;37(6):1049-50.
97. Shelley HJ. A case of uterine fibroid separated from its pedicle. Am J Surg. 1931 Dec;14(3):631-633
98. Siewert A, Kolberg A. [Uterine leiomyomata necessitating emergency postpartum hysterectomy: a case report.](https://www.ncbi.nlm.nih.gov/pubmed/18232252) S D Med. 2007 Dec;60(12):485, 487, 489.
99. Su WH, Cheung SM, Chang SP, et al. Internal bleeding from a ruptured serosal vein covering the myoma surface mimicking upper gastrointestinal bleeding. Taiwan J Obstet Gynecol 2008;47:352-354.
100. Sule AZ. Traumatic rupture of uterine fibroid: an uncommon cause of post traumatic haemoperitoneum. West Afr J Med. 2000 Apr-Jun;19(2):158-9.
101. Swarray-Deen A, Mensah-Brown SA, Coleman J. Rare complication of fibroids in pregnancy: spontaneous fibroid rupture. J Obstet Gynaecol Res 2017;43:1485-1488.
102. Tajima S, Yonezawa I, Waki M, et al. Massive hemoperitoneum following spontaneous rupture of an arterial aneurysm overlying a uterine myoma. Int J Clin Exp Med 2015;8:3002-3005.
103. [Takai H](https://www.ncbi.nlm.nih.gov/pubmed/?term=Takai%20H%5BAuthor%5D&cauthor=true&cauthor_uid=23447923), [Tani H](https://www.ncbi.nlm.nih.gov/pubmed/?term=Tani%20H%5BAuthor%5D&cauthor=true&cauthor_uid=23447923), [Matsushita H](https://www.ncbi.nlm.nih.gov/pubmed/?term=Matsushita%20H%5BAuthor%5D&cauthor=true&cauthor_uid=23447923). Rupture of a degenerated uterine fibroid as a cause of acute abdomen: a case report. [J Reprod Med.](https://www.ncbi.nlm.nih.gov/pubmed/?term=takai+fibroid+acute+abdomen) 2013 Jan-Feb;58(1-2):72-4.
104. Tan YL, Naidu A. Rare postpartum ruptured degenerated fibroid: a case report. J Obstet Gynaecol Res 2014;40:1423- 1425.
105. Tang A, S. Rao S, Cawdell G. Massive intra-abdominal haemorrhage due to spontaneous bleeding from fibroids in a postmenopausal woman. J Obstet Gynaecol. 2008 28:2, 244-245,
106. Tirelli A, Volpe A, Cagnacci A, et al. Massive Hemoperitoneum from Uterine Leiomyoma Associated with Diffuse Myometrial Venous Congestion. J Gynecol Surg 2008;24(2):79-81
107. Toquero L, Gadd R, Owers CE, et al. Post-coital haemoperitoneum: a downside to intercourse. Ann R Coll Surg Engl 2012;94:e26-e27.
108. Turner PD. Accidental rupture of pedicle of calcified fibroid. [Proc R Soc Med](/l). 1920;13(Obstet Gynaecol Sect):186–187.
109. Varras M, Antoniou S, Samara Ch, et al. [IntraperitonealHYPERLINK "https://www.ncbi.nlm.nih.gov/pubmed/12556108" haemorrhage secondary to perforation of uterine fibroid aftHYPERLINK "https://www.ncbi.nlm.nih.gov/pubmed/12556108"er cystic degeneration.HYPERLINK "https://www.ncbi.nlm.nih.gov/pubmed/12556108" Unusual CT findings resembling malignant pelvic HYPERLINK "https://www.ncbi.nlm.nih.gov/pubmed/12556108"tumorHYPERLINK "https://www.ncbi.nlm.nih.gov/pubmed/12556108": case repoHYPERLINK "https://www.ncbi.nlm.nih.gov/pubmed/12556108"rt.](https://www.ncbi.nlm.nih.gov/pubmed/12556108) Eur J Gynaecol Oncol. 2002;23(6):565-8.
110. Venables CW, Craft IL. [HaemoperitoneumHYPERLINK "https://www.ncbi.nlm.nih.gov/pubmed/6038373" after traumatic avulsion of uterine fibroid.](https://www.ncbi.nlm.nih.gov/pubmed/6038373) Br Med J. 1967 Sep 16;3(5567):723-4.
111. Von Son RN, Sedlis A. Massive hemorrhage in fibroid with cystic degeneration. N Y State J Med. 1965 Dec;65(23):2938-2940
112. Wallace AJ. Intraperitoneal Haemorrhage in Cases of Fibro‐myomata of the Uterus. J Obstet Gynaecol Br Emp. 1910 Dec;18(6):357-367
113. Woodruff MF. [Intraperitoneal haemorrhage of unusual aetiology with a report of two cases.](https://www.ncbi.nlm.nih.gov/pubmed/18900995) Br J Surg. 1948 Jan;35(139):311-3
114. Wong L, Ching TW, Kok TL, et al. [Spontaneous HYPERLINK "https://www.ncbi.nlm.nih.gov/pubmed/16305710"hemoperitoneumHYPERLINK "https://www.ncbi.nlm.nih.gov/pubmed/16305710" from a uterine HYPERLINK "https://www.ncbi.nlm.nih.gov/pubmed/16305710"leiomyomaHYPERLINK "https://www.ncbi.nlm.nih.gov/pubmed/16305710" in pregnancy.](https://www.ncbi.nlm.nih.gov/pubmed/16305710) Acta Obstet Gynecol Scand. 2005 Dec;84(12):1208-9.
115. Wyckoff ET, Moawad NS, Schultheis CM, et al. A Rare Case of Spontaneous Rupture of Endometrial Stromal Sarcoma Resulting in Acute Hemoperitoneum. J Gynecol Surg 2016;32(3):197-200
